# Efficacy and Safety of Endoscopic Papillary Large Balloon Dilation for Removal of Large Bile Duct Stones in Advanced Age

**DOI:** 10.1155/2016/6568989

**Published:** 2016-10-12

**Authors:** Kook Hyun Kim, Tae Nyeun Kim

**Affiliations:** Division of Gastroenterology and Hepatology, Department of Internal Medicine, Yeungnam University College of Medicine, Daegu, Republic of Korea

## Abstract

*Objective*. Bile duct stone-related adverse events can be detrimental in the elderly. However, little is known about clinical outcomes and adverse events following endoscopic papillary large balloon dilation (EPLBD) in the elderly. The aim of this study was to evaluate the safety and feasibility of EPLBD for the removal of CBD stones in patients aged ≥ 80 years.* Methods*. A total of 204 patients who underwent EPLBD from 2006 to 2012 were retrospectively reviewed. Patients were classified into two groups (148 patients < 80 years old, Group A; 56 patients ≥ 80 years old, Group B). Endoscopic findings, clinical outcomes, and adverse events in two groups were compared.* Results*. The number of underlying chronic diseases in Group B was significantly higher than in Group A (*P* = 0.032). The rates of overall stone clearance were similar between two groups (*P* = 0.145). No significant difference with regard to post-ERCP pancreatitis between two groups was observed (*P* = 0.687). All episodes of pancreatitis had full recovery with conservative treatment. One major hemorrhage in Group A was successfully controlled endoscopically and one death caused by retroperitoneal perforation occurred in Group A.* Conclusions*. EPLBD appear to be safe and effective for CBD stone removal in patients aged ≥ 80 years.

## 1. Introduction

The number of the elderly with common bile duct (CBD) pathology is increasing with the advent of the aged society. Endoscopic retrograde cholangiopancreatography (ERCP) is less invasive than surgery, but a highly effective procedure, and has played a central role in the management of pancreatobiliary disease in patients with advanced age [1]. Given the increasing number of therapeutic ERCP procedures in the elderly patients, procedure-related mortality might be expected to be high because adverse events could exacerbate chronic concomitant diseases. Although some reports indicate therapeutic ERCP is safe even in the elderly, mortality and morbidity might be high due to the presence of cardiovascular disorders and cerebrovascular accidents [2, 3]. Furthermore, as elderly patients are likely to have a higher bleeding tendency due to the presence of underlying disorder, there was a need for an alternative to conventional way for the management of the biliary disease. Since introduction of endoscopic papillary balloon dilation (EPBD) in 1982, it has been the effective modality for removal of CBD stone. However, great concern remains about the risk of pancreatitis [4–7]. Recently, endoscopic papillary large balloon dilation (EPLBD) with small endoscopic sphincterotomy (ES) was adopted as a rescue technique for the extraction of large-caliber stones, because it can enlarge ampullary opening to enable bulky and cylindrically shaped stones to be extracted with less difficulty, even in patients with anatomical variation [8–10].

However, little is known about clinical outcomes and adverse events following EPLBD in the extremely elderly. Accordingly, we conducted this study in order to evaluate the safety and feasibility of EPLBD with or without ES for the removal of CBD stones in patients aged ≥80 years.

## 2. Methods

### 2.1. Patients

The medical records of 204 consecutive patients with bile duct stones ≥ 10 mm in diameter that underwent EPLBD with or without ES for the removal of CBD stones from August 2006 to August 2012 were retrospectively evaluated. Exclusion criteria for this study were as follows: (1) a history of prior endoscopic sphincterotomy; (2) total gastrectomy with Roux-en-Y anastomosis; (3) septic shock; (4) concomitant acute pancreatitis; (5) the spontaneous passage of stones; and (6) patients <50 years old. These 204 patients were allocated to two groups: 148 patients aged <80 years to Group A and 56 patients aged ≥80 years to Group B. Patients were carefully monitored for potential adverse events, such as bleeding, pancreatitis, and perforation for at least for 24 hours after ERCP. Previous medical history, underlying comorbid disease, ERCP results, and associated adverse events were retrospectively evaluated. The study was approved by the institutional review board of our hospital.

### 2.2. Definitions

Post-ERCP pancreatitis was defined as a serum amylase level exceeding three times the upper normal limit (>400 U/L) combined with abdominal pain after ERCP, and its severity was graded based mainly on the length of hospital stay: mild, hospitalization of 1 to 3 days after procedure; moderate, 4 to 9 days in hospital; severe, more than 10 days in hospital [11, 12]. Hyperamylasemia was defined as a serum amylase level of >400 U/L, but without any abdominal pain. Procedure related bleeding was classified as major or minor based on amounts of hemorrhage. Major bleeding was defined as hemorrhage requiring transfusion or any intervention for hemostasis and minor bleeding as mild to self-limiting hemorrhage without a fall in hemoglobin level. Postprocedural bleeding was categorized as early or delayed. Early bleeding was defined as hemorrhage during, immediately after, or within 24 hours after ERCP. Delayed bleeding, which was confirmed endoscopically, was defined as hemorrhage occurring >24 hours after procedure.

### 2.3. Endoscopic Procedures

All endoscopic procedures were performed using side-viewing endoscopes (TJF-140; Olympus Optical Corporation, Tokyo, Japan). ERCP was carried out by experienced endoscopists at a single tertiary hospital. Patients were initially sedated with midazolam (3–5 mg) and pethidine (25–50 mg) intravenously, and propofol (10–60 mg) was administered during the procedure, if needed. After intubation of the endoscope into the 2nd portion of the duodenum, anatomical variations, including the presence and size of diverticulum, were closely evaluated. All cannulations were initiated using a standard ERCP catheter or a pull-type sphincterotome. When biliary approach failed using a standard method, a precut technique using a needle knife (Boston Scientific, Cork, Ireland) was attempted as a rescue technique.

A dilating balloon catheter (CRE balloon, Boston Scientific, Cork, Ireland) was slid along a guidewire and placed at the midpoint of the balloon across the ampullary orifice. Ballooning sizes (12–20 mm) of CRE balloon catheters were determined based on stone and CBD diameter. Balloons were gradually inflated under fluoroscopic guidance with a diluted contrast medium until the waist of balloon disappeared. Once the waist was vanished, balloon inflation was maintained for 10 to 60 seconds. A mechanical lithotriptor was used to crush stones too large to retrieve intact.

When a patient was unfit for complete stone removal in a single stage, due to a higher bleeding risk or poor general condition, an endoscopic nasobiliary drainage (ENBD) tube or an endoscopic retrograde biliary drainage (ERBD) stent was placed primarily. After discontinuation of subsequent antiplatelet or anticoagulant medication for 3–5 days and an improvement in condition, 2nd look ERCP was performed (two-stage method). Stone clearance was confirmed either by cholangiogram or by using an ENBD tube.

### 2.4. Statistical Analysis

Statistical analysis was conducted using Student's *t* test and the chi-square test in SPSS version 17.0 (SPSS, Inc., Chicago, IL, USA). A *P* value of <0.05 was considered statistically significant.

## 3. Results

Demographic characteristics of the 204 patients are summarized in Table 1. The mean age was 68.5 ± 7.4 years (range, 50–79) in Group A (<80 yrs) and 84.5 ± 3.1 years (range, 80–92) in Group B (≥80 yrs). The male ratio was significantly higher in Group A (60.1% versus 32.1%, *P* < 0.001), and the frequency of EPLBD with limited ES was significantly lower in Group A (54.1% versus 64.3, *P* < 0.001). Twenty-nine (19.6%) patients in Group A had a history of Billroth II gastrectomy and 6 patients (10.7%) in Group B (*P* = 0.133). The number of underlying chronic diseases was significantly lower in Group A (49.3% versus 66.1%, *P* = 0.032), especially dementia (0% versus 10.7%, *P* < 0.001). However, no significant intergroup differences with regard to cerebrovascular accidents, hypertension, coronary artery disease, congestive heart failure, end stage renal disease, liver cirrhosis, and chronic respiratory disease were observed. Although the numbers of periampullary diverticulum were similar between two groups, the frequency of large diverticulum was significantly higher in Group B (23.2% versus 8.8%, *P* = 0.006). Mean stone size and bile duct diameter were not significantly different between two groups (15.6 ± 5.6 mm versus 17.1 ± 6.0 mm, *P* = 0.094, and 19.4 ± 5.1 mm versus 21.0 ± 5.5 mm, *P* = 0.052, resp.).

Clinical outcomes of EPLBD in the 204 study subjects are described in Table 2. Complete stone removal was performed in 96.1% (196/204), and rates of overall stone clearance and stone retrieval in first sessions were not significantly different in the two groups (97.3% versus 92.9%, *P* = 0.145; and 68.2% versus 75.0%, *P* = 0.682, resp.). No significant intergroup differences were observed regarding the frequency of mechanical lithotripsy, pancreatography, and biliary stenting. Procedure-related adverse events are listed in Table 3. Of the early procedure-related adverse events, rates of post-ERCP pancreatitis, acute cholangitis, bleeding, and perforation were not significantly different between Group A and Group B [5/148 (3.4%) versus 3/56 (5.4%), *P* = 0.687; 2/148 (1.4%) versus 0, *P* = 1.000; 3/148 (2.1%) versus 0%, *P* = 0.313; 1/148 (0.7%) versus 0%, *P* = 1.000, resp.]. All episodes of pancreatitis were completely recovered by conservative treatment. Of the 3 cases (2.1%) of bleeding, one was major and 2 were minor. One major hemorrhage in Group A requiring 5 pints of blood transfusion was successfully controlled by a series of endoscopic treatments, with no further consequence. One death occurred in Group A due to a retroperitoneal perforation after EPLBD.

## 4. Discussion

Pancreatobiliary tract diseases, including choledocholithiasis, are frequently encountered in the elderly [3], and bile duct stone-related adverse events can be detrimental in elderly patients because of the high prevalence of comorbidities, such as cardiopulmonary or cerebrovascular disease. In particular, the elderly are susceptible to procedure-associated infections, such as cholangitis or cholecystitis, due to the weak immune system, which may largely explain higher rates of morbidity and mortality in the elderly [13].

Recently, the advent of EPLBD made it possible to remove bulky stones in the clinical setting. However, although EPLBD has reduced the need for mechanical lithotripsy, its use requires discretion because its safety has not been fully established, particularly, in patients older than 80 [14]. Elderly patients have significantly higher rates of underlying disorders than young patients (66.1% versus 49.3%, *P* = 0.032), and in the present study, this was particularly true of dementia (*P* < 0.001), as has been previously reported [15–17]. Recent studies have found that PAD exhibits an increasing propensity with age [18, 19], and in the present study, the incidence of PAD was higher in the elderly group (60.7% versus 45.9%, *P* = 0.060) and the frequency of large-sized PAD is considerably higher in elderly group (23.2% versus 8.8%, *P* = 0.006). Biliary cannulation is challenging due to compression by huge diverticula [20], but after deep cannulation has been accomplished, the ampullary orifice abutting PAD can be easily dilated with a balloon dilator. However, despite the wide use of EPLBD in patients with PAD, the risks of perforation are of critical concern for endoscopists [21, 22].

In the present study, rates of overall CBD stone clearance and successful stone removal in first ERCP sessions did not differ significantly between the young and elderly groups (97.3% versus 92.9%, *P* = 0.145 and 68.2% versus 75.0%, *P* = 0.682, resp.), which concurs with a prior report [15]. Mechanical lithotripsy is an indispensable technique for successful removal of the complicated stones. A series of recent studies reported EPLBD with or without ES required mechanical lithotripsy less often than ES [21, 22]. In the present study, no significant intergroup difference was found in terms of the need for mechanical lithotripsy (*P* = 0.672).

Of the early adverse events after ERCP, pancreatitis, bleeding, and perforation are the most significant adverse events associated with EPLBD. Recent studies showed the post-ERCP complication rates in the elderly patients were similar to that in the general population, ranging from 2.5% to 4.7% [1, 3]. Previous studies have reported that adverse events rates in elderly patients range from 2.9% to 6.8% [15, 17].

Post-ERCP pancreatitis is one of the most terrifying adverse events. Several hypotheses have been proposed for the mechanism of post-ERCP pancreatitis. First, the pancreatic duct can be physically compressed during ballooning, and this temporarily hampers pancreatic fluid flow through the ampullary orifice. Several authors have suggested ES prior to EPLBD might provide a solution to the development of pancreatitis after EPLBD because minimal ES could partially alleviate the pressure burden on the pancreatic orifice [8, 23, 24]. Second, several failed attempts of cannulation, which causes mucosal edema due to direct injury of the pancreatic orifice, are considered the primary cause of pancreatitis, particularly, in patients with a huge diverticulum because the distal CBD is anatomically squeezed and displaced. In the present study, although the elderly group had a higher prevalence of a large diverticulum, rates of postprocedural pancreatitis were similar in the two groups (*P* = 0.687), which concurs with a recently published study [15]. The incidence of post-ERCP pancreatitis in our elderly group (5.4%) was in the acceptable range, but it was slightly higher than the previously published data (0.8%–2.3%) [3, 15, 16, 23, 25]. It has been reported that the rate of pancreatitis in the elderly is comparatively low because exocrine function is diminished due to atrophy of pancreatic acinar tissue [26]. Interestingly, CBD diameter in our elderly group was greater than in the young group, which concurs with the suggestion that long-lasting floating ductal stones can make the ampullary orifice patulous and reduce the risk of pancreatitis [27]. Bleeding was one of the serious adverse events associated with ERCP. The present study revealed no significant difference with regard to bleeding rate between the two groups (2.1% versus 0%, *P* = 0.313), which was comparable with previous studies [27–29]. In a previous study, diverticulum was found to be a significant risk factor of post-ERCP bleeding [23]. In the present study, of the 3 cases (2.1%) of bleeding encountered, a case of major bleeding occurred in a patient with CVA following small ES prior to EPLBD. A number of recent studies have shown EPLBD alone can be an option, especially in patients with a coagulopathy, because it can lower the bleeding rate (0–2.4%) by avoiding ES, with similar therapeutic outcomes, compared with EPLBD with ES (3.3–10.0%) [15, 23, 30].

Perforation is the most life-threatening adverse event in the elderly. A recent article identified distal biliary stricture as an independent risk factor of perforation [31]. In the present study, we experienced one episode (0.4%) of perforation. Theoretically, radial force exerted around the surface of the balloon can cause the weakest region of the ampullary mucosa to rupture during the balloon manipulation. For successful EPLBD, proper ballooning size and gradual, cautious dilation are of the utmost importance [32].

Considering the high rate of mortality and morbidity after the surgery in elderly patients, ERCP is an alternative for the management of choledocholithiasis in such patients [1]. The present study supports previous studies regarding the feasibility of EPLBD in the elderly patients. However, this retrospective study was conducted at a tertiary hospital and had the following limitations: (a) lack of adequate duration of ballooning dilation and (b) lack of definite indications for EPLBD or EPLBD with ES. Hence, large randomized prospective studies are required to elucidate the clinical effectiveness of EPLBD in the elderly patients. It is suggested that careful selection of candidates and the highly experienced endoscopists will lower the adverse events rate and achieve the favorable outcomes in the elderly.

## Figures and Tables

**Table 1:** Demographics and baseline characteristics.

Variables	Total (*n* = 204)	<80 yr (*n* = 148)	≥80 yr (*n* = 56)	*P* value
Age (years, range)	73.0 ± 9.6 (50–92)	68.5 ± 7.4 (50–79)	84.5 ± 3.1 (80–92)	<0.001
Sex (M)	107 (52.5)	89 (60.1)	18 (32.1)	<0.001
BMI (kg/m^2^)	22.4 ± 3.2	22.5 ± 3.1	22.0 ± 3.4	0.261
With ES	116 (56.9)	80 (54.1)	36 (64.3)	<0.001
Precutting	28 (13.7)	22 (14.9)	6 (10.7)	0.442
Previous surgery				
Cholecystectomy	29 (14.2)	21 (14.2)	8 (14.3)	0.986
Billroth II gastrectomy	35 (17.2)	29 (19.6)	6 (10.7)	0.133
Underlying chronic disease	110 (53.9)	73 (49.3)	37 (66.1)	0.032
Neurologic				
CVA	20 (9.8)	17 (11.5)	3 (5.4)	0.291
Dementia	6 (2.9)	0	6 (10.7)	<0.001
Hypertension	84 (41.2)	56 (37.8)	28 (50.0)	0.115
Cardiovascular				
Coronary heart disease	12 (5.9)	7 (4.7)	5 (8.9)	0.255
Congestive heart failure	9 (4.4)	6 (4.1)	3 (5.4)	0.708
Chronic renal failure	5 (2.5)	4 (2.7)	1 (1.8)	1.000
Liver cirrhosis	7 (3.4)	7 (4.7)	0	0.193
COPD	9 (4.4)	4 (2.7)	5 (8.9)	0.118
Periampullary diverticulum	102 (50.0)	68 (45.9)	34 (60.7)	0.060
Large diverticulum (>3 cm)	26 (12.7)	13 (8.8)	13 (23.2)	0.006
Gallstones	85 (41.7)	60 (40.5)	25 (44.6)	0.596
CBD stones				
Number (1/2/≥3)	101/33/70	75/25/48	26/8/22	0.434
Size of stones (mm, range)	16.0 ± 5.7 (10–37)	15.6 ± 5.6 (10–35)	17.1 ± 6.0 (10–37)	0.094
Type (brown/black/cholesterol)	38/166/204	131/132/1	47/7/1	0.285
CBD pathology				
CBD diameter (mm, range)	19.8 ± 5.3 (9.1–35.5)	19.4 ± 5.1 (9.1–35.5)	21.0 ± 5.5 (10.2–35.2)	0.052
Distal CBD stricture	11 (5.4)	8 (5.4)	3 (5.4)	0.989
Size of balloon dilator (mm, range)	15.4 ± 2.4 (12–20)	15.3 ± 2.4 (12–20)	15.7 ± 2.3 (12–20)	0.275
Duration of balloon (mm, range)	38.0 ± 16.1 (10–60)	38.6 ± 16.7 (10–60)	36.3 ± 14.5 (10–60)	0.321

BMI: body mass index; ES: endoscopic sphincterotomy; CVA: cerebrovascular accident; COPD: chronic obstructive pulmonary disease; CBD: common bile duct. Values are presented as mean ± SD (range) or as numbers (%).

**Table 2:** Clinical outcomes of endoscopic retrograde cholangiopancreatography.

Variables	Total (*n* = 204)	<80 years (*n* = 148)	≥80 years (*n* = 56)	*P* value
Overall stone clearance	196 (96.1)	144 (97.3)	52 (92.9)	0.145
Number of sessions of endoscopy				
1	143 (70.1)	101 (68.2)	42 (75.0)	0.682
2	51 (25.0)	41 (27.7)	10 (17.9)	
≥3	10 (4.9)	6 (4.1)	4 (7.1)	
Stent placement				
ENBD	38 (18.6)	27 (18.2)	11 (19.6)	0.819
ERBD	33 (16.2)	24 (16.2)	9 (16.1)	0.980
Mechanical lithotripsy	19 (9.3)	13 (8.8)	6 (10.7)	0.672
Pancreatogram	46 (22.5)	36 (24.3)	10 (17.9)	0.324

ENBD: endoscopic nasobiliary drainage; ERBD: endoscopic retrograde biliary drainage. Values are presented as numbers (%).

**Table 3:** Postprocedural adverse events between two groups.

Variables	<80 years (*n* = 148)	≥80 years (*n* = 56)	*P* value
Total	9 (6.1)	3 (5.4)	1.000
Pancreatitis	5 (3.4)	3 (5.4)	0.687
Mild	4 (80.0)	2 (66.7)	
Moderate	1 (20.0)	1 (33.3)	
Acute cholangitis	2 (1.4)	0	1.000
Acute cholecystitis	1 (0.7)	0	1.000
Bleeding	3 (2.1)	0	0.313
Major	1 (0.7)	0	
Minor	2 (1.4)	0	
Perforation	1 (0.7)	0	1.000
Mortality	1 (0.7)	0	1.000

Values are presented as numbers (%).
